# Cervical Septal Remnants

**Published:** 2012-12-17

**Authors:** Firozeh Ahmadi, Hadieh Haghighi

**Affiliations:** Department of Reproductive Imaging, Reproductive Biomedicine Research Center, Royan Institute for Reproductive Biomedicine, ACECR, Tehran, Iran

**Fig 1:**
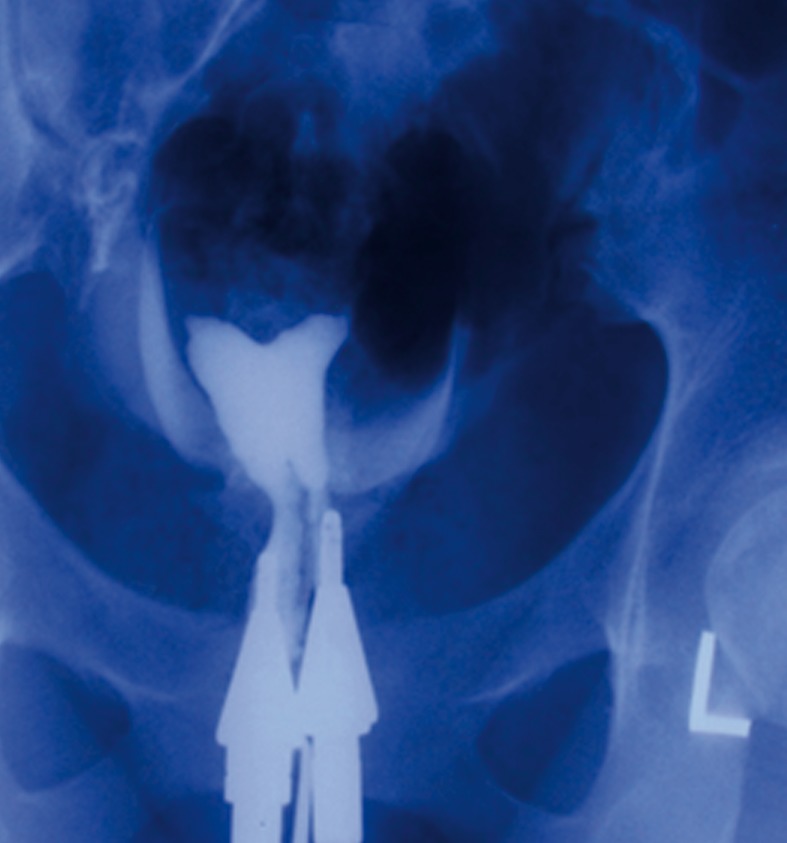
Hysterosalpingography (HSG) after surgery revealed an arcuate uterus with two cervical canal.

A 30-year-old woman with a history of two pregnancy losses in the 8^th^ and 16^th^ weeks of pregnancy and a history of metroplasty referred to our Infertility Clinic. Hysterosalpingography (HSG) was performed as a standard infertility evaluation following surgery (Fig 1).

**Fig 2:**
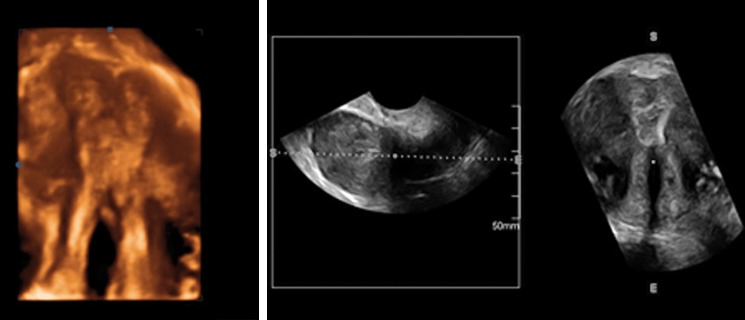
3-D ultrasound image after metroplasty.

**Fig 3:**
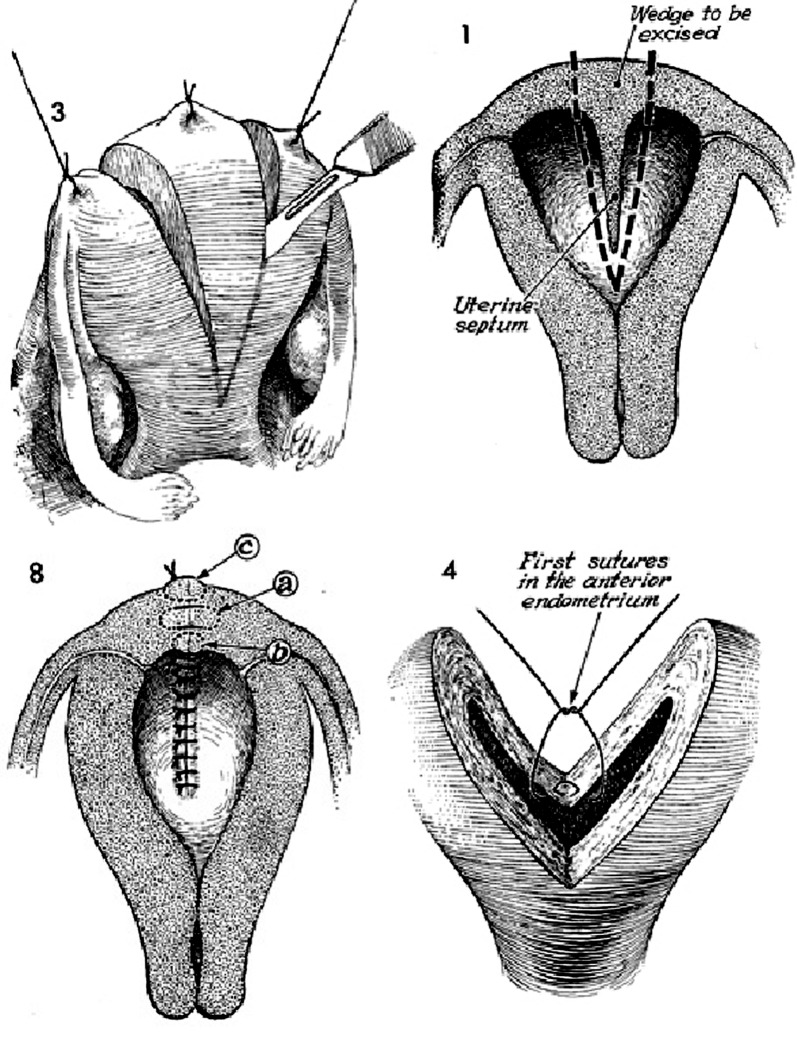
Dissection of septum from fundus via the Jones procedure results in a relatively irregular fundal appearance.

This was a case of longitudinal septate uterus which began at the fundus of the uterus and extended to the external os in the cervix. The septum was surgically resected in the endometrial cavity however we left the septum of the cervix intact (Fig 2).

Early in female embryonic development the uterus, oviduct, and vagina arise from paired Mϋllerian structures which finally fuse at midline. Müllerian duct anomalies (MDAs) result from defects in fusion or incomplete resorption. Failure of resorption of the median septum results in a septate or subseptate uterus. Complete or partial duplication of the uterine cavities depends on whether the failure of resorption is complete or partial (1 ).

HSG has been used as a routine test to assess the uterine cavity as either an initial or post-operative investigation.

Three dimensional (3-D) ultrasound is a valid alternative to HSG in the diagnosis of MDAs, particularly for evaluation of patients who have had a history of uterine surgery. The relationship between the fundus and the uterine cavity can be depicted perfectly with sonographic reconstructions in the coronal plane, and it can assist with measuring the length and thickness of the septum. These characteristics of the septum are significant factors in preoperative decision-making. In this case, we have additionally obtained images by 3-D vaginal ultrasound.

As shown in figure 2 there was a complete match between HSG and 3-D ultrasound images after surgery.

Congenital MDAs are estimated to affect 0.16%- 10% of the general population (1 ). The septate uterus, which is the most common form of MDAs is associated with the highest rate of pregnancy failure. Complete septate uterus with cervical duplication and longitudinal vaginal septum is a developmental failure of resorption of the median septum (2 ).

Septate uterus can be treated by various surgical techniques. Until the mid-1980s, surgeons performed transabdominal metroplasty (Strassman, Tompkins, and Jones operations) to treat septate uterus (Fig 3). Despite differences in the surgical details, these three procedures necessitated excision of the entire septum by a wedge resection which involved bivalving the uterus in an anteroposterior direction (3 , 4 ).

In recent years, operative hysteroscopy offers a new method of hysteroscopic metroplasty with effective, safe results. In this method the septum is removed by an incision rather than an excision (2 , 5 ). When dealing with infertility, the early diagnosis of MDAs and precise post-operative investigation are both crucial for optimal management of patients.

In conclusion, a 3-D ultrasound should be considered as the method of choice if there is a suspicion of Müllerian anomalies or for post-operative investigation. Advantages of this technique are its high diagnostic accuracy, it is easy to perform, and has less complications due to X-ray exposure and iodine injection, which is inevitable for HSG. A high accordance has been reported between the findings of 3-D ultrasounds and magnetic resonance imaging in the diagnosis of MDAs (6 ).
